# Stature and mitigation systems affect the risk of leg injury in vehicles attacked under the body by explosive devices

**DOI:** 10.3389/fbioe.2023.918013

**Published:** 2023-02-03

**Authors:** Eduardo A Rebelo, Grigoris Grigoriadis, Diagarajen Carpanen, Anthony M. J. Bull, Spyros Masouros

**Affiliations:** Department of Bioengineering, Imperial College London, London, United Kingdom

**Keywords:** blast injury, anthropometry, combat boots, finite element modeling, foot and ankle, high loading rate, protection, mitigation

## Abstract

A finite-element (FE) model, previously validated for underbody blast (UBB) loading, was used here to study the effect of stature and of mitigation systems on injury risk to the leg. A range of potential UBB loadings was simulated. The risk of injury to the leg was calculated when no protection was present, when a combat boot (Meindl Desert Fox) was worn, and when a floor mat (IMPAXX^TM^), which can be laid on the floor of a vehicle, was added. The risk of injury calculated indicates that the floor mat provided a statistically significant reduction in the risk of a major calcaneal injury for peak impact speeds below 17.5 m/s when compared with the scenarios in which the floor mat was not present. The risk of injury to the leg was also calculated for a shorter and a taller stature compared to that of the nominal, 50th percentile male anthropometry; shorter and taller statures were constructed by scaling the length of the tibia of the nominal stature. The results showed that there is a higher risk of leg injury associated with the short stature compared to the nominal and tall statures, whereas the leg-injury risk between nominal and tall statures was statistically similar. These findings provide evidence that the combat boot and the floor mat tested here have an attenuating effect, albeit limited to a range of possible UBB loads. The effect of stature on injury has implications on how vehicle design caters for all potential anthropometries and indeed gender, as women, on average, are shorter than men. The results from the computational simulations here complement laboratory and field experimental models of UBB, and so they contribute to the improvement of UBB safety technology and strategy.

## 1 Introduction

In recent conflicts, explosive devices have caused damage to numerous land vehicles and resulted in a range of injuries to their occupants (Kempinski and Murphy, 2012; McGuire et al., 2019). The transmission of the blast loading through the vehicle, primarily through the floor, termed underbody blast (UBB), can result in difficult-to-treat ankle, foot, and leg injuries that present significant morbidity (Asensio and Trunkey, 2008; Ramasamy et al., 2013).

In order to minimize the severity of these blast injuries, mitigation strategies have included the targeted redesign of armored vehicles and the development of mobile personal protection systems (Ramasamy et al., 2009; Kamel, 2019). An energy-dissipating structure that has been used between the encroaching floor and the occupant’s feet is a floor mat; indeed, it has been shown in physical experiments that floor mats are able to attenuate load under impact (Newell et al., 2013).

Furthermore, whilst in the civilian automotive setting it is known that stature and gender may affect the severity of leg injury (Crandall et al., 1996; Welsh et al., 2003; Malczyk et al., 2013; Hu et al., 2017; Ammori and Abu-Zidan, 2018), there have been no such studies for UBB. Stature can vary substantially amongst vehicle occupants; the height of 180 American soldiers was found to vary between 1.53 and 1.76 m for women and 1.52 and 1.93 m for men (Darter et al., 2012).

The use of anthropomorphic test devices (ATDs) is limited in terms of both relevance to UBB loading and variability in stature alike, and experimental testing with cadaveric tissue is limited in terms of the number of tests that can be conducted across a range of potential UBB loading. Computational modeling, however, offers a realistic alternative to simulating UBB. Therefore, this study aimed to use a validated lower limb finite-element (FE) model of UBB to quantify the protection offered by vehicle-design mitigation represented by a floor mat and assess the influence of stature on injury risk.

## 2 Methods

**FIGURE 1:**
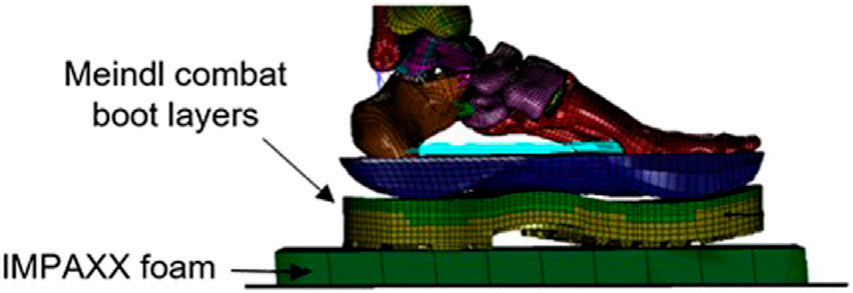
Distal part of the finite-element model developed previously by Rebelo et al. (2021) and used here to study the effect of the IMPAXX floor mat and stature on injury.

An FE model of the lower limb developed in PATRAN (v2018, MSC. Software, Santa Ana, CA, United States), solved in Dytran (v2018, MSC. Software, Santa Ana, CA, United States), and validated for UBB-related loading conditions was used (Rebelo et al., 2021) (Figure 1). Briefly, the FE model is of a cadaveric lower limb with anthropometry close to that of the 50th percentile American male (height = 1.727 m; weight = 72.6 kg). The model response was compared against that of laboratory-based UBB tests conducted at two independent laboratories. The computational signals were found to be within the experimental corridors and, when experimental corridors were not available, acceptably close in magnitude to values reported in these studies. A version of the validated model incorporated a combat boot, specifically a UK-size 10 Meindl Desert Fox combat boot (Lukas Meindl GmbH & Co., Germany). Briefly, the combat boot used is made up of three layers, namely, insole, midsole, and outsole, and a cardboard insert that structurally supports the insole and allows integration with the lower layers. Simulations were run across a range of UBB-related loading conditions, and the amount of protection offered by the boot was quantified.

Here, two sets of UBB simulations were set up. The first one incorporated the IMPAXX™ foam between the loading plate representing the vehicle’s floor and the sole of the combat boot in the original, baseline model. IMPAXX™ is a commercial foam available for use in vehicle floors as a floor mat. It is a closed cell foam, which was modeled using the FOAM2 material model and a material stress–strain field (Slik et al., 2006).

**FIGURE 2:**
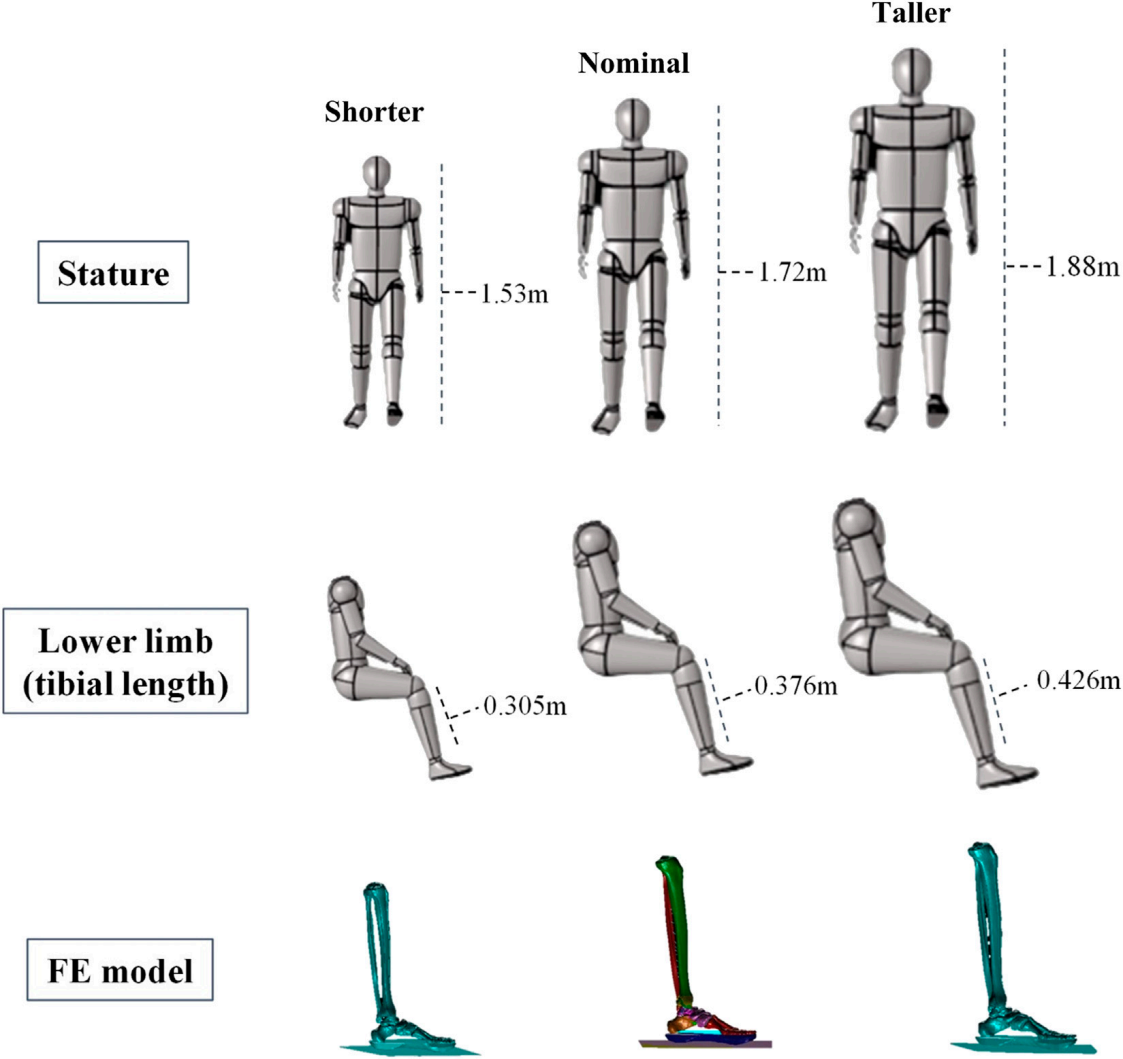
Short, nominal, and tall statures used for the anthropometric study. They were selected based on the military stature interval reported by Darter et al. (2012).

The second set of simulations was of two statures representing a shorter and a taller stature to that used in the baseline model, which was considered to be a medium, average stature (tibia length = 386 mm) (Figure 2). The objective was to assess the effect of stature alone on the risk of foot-and-ankle injury. In order to obtain the geometry of the short and tall statures, the lower limb of the baseline, medium stature model was scaled. The length of the tibia was used for scaling as it has been shown to correlate well with stature (Duyar and Pelin, 2003). Scaling was achieved in HyperMesh (Altair, v2014, CA, United States) using the relationship established by Duyar and Pelin (2003). All meshes were morphed linearly with the same scaling factor. The relative position between components (bones) was kept, and the cortical thickness of all tarsal bones remained unaltered. All other model parameters, including the material properties, were not changed. The combat boot and floor mat were included in all simulations.

UBB-representative vehicle-floor velocity profiles with peak velocities ranging from 5 to 17.5 m/s and time to peak between 1.5 and 9 ms were applied to the loading plate (Rebelo et al., 2021) (Supplementary Table S1). These profiles were triangular in shape, consisting of a section in which the loading velocity increased until the peak velocity reached at the time to peak velocity and a second section in which the velocity decreased to zero with the same slope as it increased to peak velocity. The peak force at the proximal end of the tibia was the output variable of interest; this was used to calculate the risk of injury based on the injury risk curve of Chirvi et al. (2017). Additionally, deformation was monitored in order to quantify the deformation pattern of the foot-and-ankle complex.

The student’s *t*-test was used to compare between groups. The statistical analysis was performed in MATLAB (R2019a, MathWorks Inc., Natick, MA) using a script that included the *t* test2 (x,y) function. A two-sample *t*-test was conducted to verify whether the means of normal distributions were equal for a 5% significance level.

## 3 Results

**FIGURE 3:**
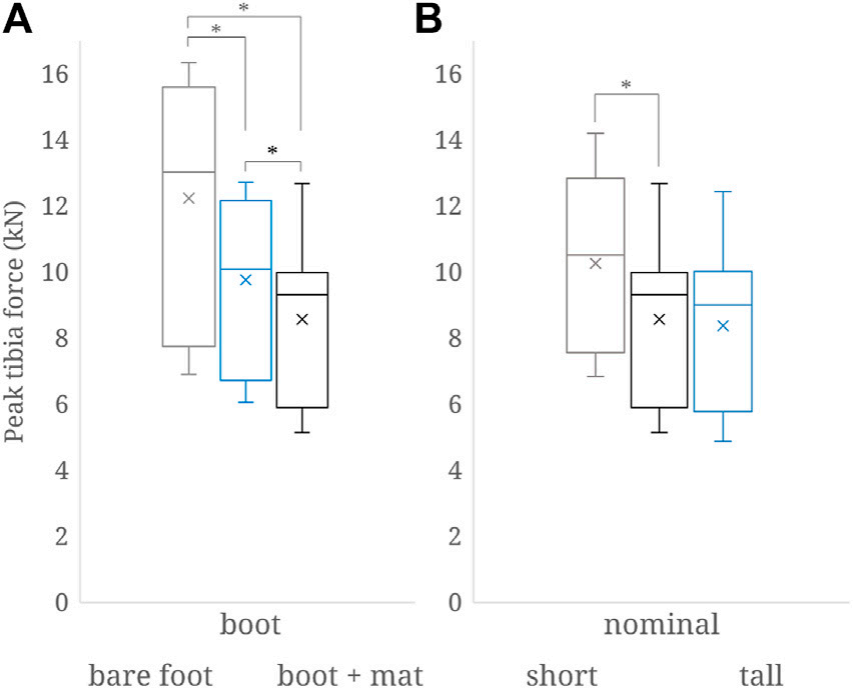
Comparison of peak tibia force distributions between **(A)** original, baseline model and mitigation represented by the combat boot (data from (Rebelo et al., 2021)) and the combat boot and the IMPAXX floor mat; **(B)** short, tall, and the nominal stature all with a combat boot and the floor mat. Statistical significance (*p* < .05) using the Student’s t-test between the distributions is noted with the asterisk.

The normal density distribution function fits of peak tibial force response for the bare foot, combat boot, and combat boot with IMPAXX™ models were significantly different from one another (Figure 3A). The normal density distribution function fit for the smaller stature was shown to be significantly different from both baseline and taller statures (Figure 3B), whereas baseline and taller statures were statistically similar. All resultant forces and associated calcaneal injury risks predicted by the simulations and used to generate Figure 3 are available in Supplementary Table S1.

The compression of the combat boot layers can be used as a surrogate marker for the resulting severity of the insult (see Supplementary Figures S1, S2). For acceleration profiles with a peak impact speed of 11.25 m/s, there was a straightening of the combat boot layers, and the layer compression at the heel site was at the order of 16% at 7 m/s. For acceleration profiles with a peak impact speed of 17.5 m/s, the compression of the combat boot layers at the heel site was 43%; the calcaneus translated distally, and plantar tissue deformation was observed. In the simulation case including the IMPAXX™ floor mat, limited deformation to the combat boot structure was observed along with less compression of the combat boot layers when compared with the simulations without the floor mat for a wide range of loading inputs.

## 4 Discussion

This study explored the effect of stature, a floor mat, and a combat boot on leg injury risk across a range of UBB loading using a previously developed FE model validated for UBB-relevant loading.

The incorporation of the Meindl combat boot resulted in a decrease between 18% and 24% in the force transmitted to the leg for peak impact speeds above 11.25 m/s, which is within the range of previous studies that explored loading attenuation to the tibia from the use of combat boots (Manseau and Keown, 2005; Geurts et al., 2006; North Atlantic Treaty Organisation RTO-TR-HFM-090, 2007; Newell et al., 2012; Kamel, 2019).

Longer times to peak were found to be associated with a larger floor-mat compression and a reduction in the force transmitted to the leg. Although the peak forces were reduced with this protective system in comparison with the configuration without it, the specific floor mat did not offer an effective reduction in injury risk for loading with peak velocities above 11.25 m/s.

The response of the FE model had been shown previously in comparison with that from physical experiments across a range of UBB-loading encompassing anatomical variability (Rebelo et al., 2021). The introduction of the IMPAXX™ floor mat in the model expectedly changed the resulting response; albeit there are no experimental data against which comparisons can be made, the response did not change to an extent that would render the model invalid. This gives confidence that the observations with the incorporation of the IMPAXX™ floor mat are reasonable. A limitation is that the representation of the IMPAXX™ floor mat is simplified to maintain efficient simulation run times. This simplification is likely to have resulted in an underestimation of load attenuation since it does not account for all cellular deformation modes and their effects on energy absorption.

The results of this study are specific to the type and model of the combat boot and floor mat used. It is likely that there are differences in levels of attenuation between different types of combat boot and floor mat. The FE model used here can be utilized to establish the level of attenuation of specific designs of the combat boot or floor mat across the range of UBB loading.

The findings of this study indicate that there is a higher risk of calcaneal injury associated with short statures than medium and tall statures (Figure 3B). Force data for the tall stature are slightly, but not statistically, significantly higher than those of the medium stature. There was, however, a reduction in injury risk for a major calcaneal injury across all loading cases for the taller stature (Supplementary Table S1). There are no epidemiological data on appropriate resolution in military populations against which one could compare these findings. Short statures and some very tall statures, however, have been shown to be of higher risk of lower leg injury than a medium stature in road-traffic frontal collisions in epidemiological (Dischinger et al., 1995; Crandall et al., 1996; Welsh et al., 2003; Austin 2012) and in computational studies (Hu et al., 2017). When appreciating the differences between military and civilian vehicle seating, loading direction, and loading rate, the effect of stature on the risk of lower leg injury found in this study is similar to that reported for road-traffic frontal collisions.

This study used the force at the proximal tibia as the metric, or injury criterion, to quantify the risk of injury to the calcaneus, according to the curves by Chirvi et al. (2017) developed from cadaveric experiments. Although other metrics could have been considered, such as strain in the calcaneus, the data by Chirvi et al. (2017) are the most reliable to date that are specific to UBB loading. Furthermore, the small change in long-bone cortical thickness during morphing to generate the taller and shorter statures would not have affected the force measured at the proximal tibia; this, combined with the fact that cortical thickness in the tarsal bones and material properties remained unaltered, means that the differences in injury risk seen between statures can be attributed to the changes in stature alone.

The influence of stature on the risk of UBB injury can be considered in the context of assessing injury tolerance for men compared to women in UBB. It is well established that women have, on average, shorter statures than their male counterparts (Duyar and Pelin, 2003) and so, based on the findings here, are at a higher risk of leg injury. This corroborates with road-traffic frontal collision research (Dischinger et al., 1995; Crandall et al., 1996; Welsh et al., 2003; Austin 2012, Ammori and Abu-Zidan, 2018); Austin (2012) reported 4–7 times higher odds for female drivers to present a leg fracture, and Dischinger et al. (1995) reported a 20% risk of lower limb fractures for females compared to 13% for male drivers with most injuries sustained at the foot and ankle; they also report that, when grouped by stature, the smaller statures were at a higher risk of leg injury than taller statures. Of course, there are biological and anatomical differences between genders other than stature that may contribute to differences in the risk of lower leg injury. The injury–risk curve used in this study to assess the risk of calcaneal fracture had been developed using male specimens exclusively (Chirvi et al., 2017). Cadaveric studies that have included gender as a covariate, such as that of Mildon et al. (2018) on the risk for a foot and ankle fracture in UBB, have all shown that, on average, women have a lower tolerance to injury than men.

Despite no relevant epidemiological data on women in the military, to date, to corroborate laboratory findings, the overwhelming evidence of experimental and computational UBB injury models is that women are at a higher risk of foot and ankle injury than men. This, combined with the findings of road-traffic frontal collision research, to date, supports that military-vehicle design mitigation strategies should consider stature as a risk factor.

In summary, a validated FE model was used to investigate the effect of the combat boot, floor mat, and stature on the risk of foot and ankle injury in UBB. The findings suggest that shorter individuals are at a higher risk of leg injury than taller individuals in UBB. A combat boot and a floor mat were shown to have a variable attenuating efficiency depending on the UBB loading characteristics, but they were shown to reduce the risk of leg injury for UBB loading with a peak floor velocity under 17.5 m/s. The versatility and efficiency of the FE model compared to expensive and laborious experimental tests render it a powerful tool toward improving UBB safety technology and strategy.

## Data Availability

The original contributions presented in the study are included in the article/Supplementary Material; further inquiries can be directed to the corresponding author.

## Supplementary material

Supplementary Figure S1 Deformation plots for simulation configurations **(A)** unreformed, **(B)** sim 1; 5 m/s in 1.5 ms, **(C)** sim 6; 11.5 m/s in 1.5 ms, and **(D)** sim 11; 17.5 m/s in 1.5 ms, all with the combat boot and without the floor mat.

Supplementary Figure S2 Deformation plots for simulation configurations **(A)** unreformed, **(B)** sim 1; 5 m/s in 1.5 ms, **(C)** sim 6; 11.5 m/s in 1.5 ms, and **(D)** sim 11; 17.5 m/s in 1.5 m/s, all with the combat boot and the IMPAXX™ floor mat.

Supplementary Table S1 Raw data per simulation with which Figure 3 was produced. The input in the simulation was a triangular pulse defined by the maximum velocity (vmax) and the time to maximum velocity or time to peak (TTP). The output from the simulations was the tibial peak force (Fmax). The peak force was then used to look up the risk of a minor or a major calcaneal injury on Chirvi et al.’s injury risk curve.
